# Data Integration and Analytics in the Dairy Industry: Challenges and Pathways Forward

**DOI:** 10.3390/ani15030329

**Published:** 2025-01-24

**Authors:** Victor E. Cabrera, Jeffrey Bewley, Mitch Breunig, Tom Breunig, Walt Cooley, Albert De Vries, Robert Fourdraine, Julio O. Giordano, Yijing Gong, Randall Greenfield, Haowen Hu, Andy Lenkaitis, Mutian Niu, Eduardo A. F. Noronha, Michael Sullivan

**Affiliations:** 1 Department of Animal and Dairy Sciences, University of Wisconsin—Madison, Madison, WI 53706, USA; gong44@wisc.edu; 2 Holstein Association USA, Brattleboro, VT 05301, USA; jbewley@holstein.com; 3 Mystic Valley Dairy, LLC, Mazomanie, WI 53560, USA; mysticvalley336@gmail.com; 4 Independent Researcher, Middleton, WI 53562, USA; jump-start@outlook.com; 5 AgProud Publishing, Jerome, ID 83338, USA; walt.cooley@agproud.com; 6 Department of Animal Sciences, University of Florida, Gainesville, FL 32611, USA; devries@ufl.edu; 7 Dairy Records Management Systems, Raleigh, NC 27603, USA; rhfourdr@ncsu.edu; 8 Department of Animal Science, Cornell University, Ithaca, NY 14853, USA; jog25@cornell.edu (J.O.G.); hh598@cornell.edu (H.H.); 9 Vita Plus Corporation, Madison, WI 53713, USA; rgreenfield@vitaplus.com; 10 GEA, St Charles, IL 60174, USA; andy.lenkaitis@gea.com; 11 Animal Nutrition, Department of Environmental Systems Science, Institute of Agricultural Sciences, ETH Zürich, 8092 Zürich, Switzerland; mutian.niu@usys.ethz.ch; 12 Informatic Department, Federal Institute of Goiás, Goiâna 74690-900, Brazil; eduardo.freitas@ifg.edu.br; 13 IYOTAH! Solutions, Westminster, CO 80030, USA; michael.sullivan@iyotah.com

**Keywords:** data integration, dairy farming, standardization, decision support systems, sustainability

## Abstract

The dairy industry faces significant challenges in data integration and analysis, which are critical for informed decision-making, operational optimization, and sustainability. Data integration—combining data from diverse sources, such as herd management systems, sensors, and diagnostics—remains difficult due to the lack of standardization, infrastructure barriers, and proprietary concerns. This commentary explores these issues based on insights from a multidisciplinary group of stakeholders, including industry experts, researchers, and practitioners. Key challenges discussed include the absence of a national animal identification system in the US, high IT resource costs, reluctance to share data due to competitive disadvantages, and differences in global data handling practices. Proposed pathways forward include developing comprehensive data integration guidelines, enhancing farmer awareness through training programs, and fostering collaboration across industry, academia, and technology providers. Additional recommendations involve improving data exchange standards, addressing interoperability issues, and leveraging advanced technologies, such as artificial intelligence and cloud computing. Emphasis is placed on localized data integration solutions for farm-level benefits and broader research applications to advance sustainability, traceability, and profitability within the dairy supply chain. These outcomes provide a foundation for achieving streamlined data systems, enabling actionable insights, and fostering innovation in the dairy industry.

## Simple Summary

Dairy farming generates a vast amount of data from different sources, such as sensors, herd management software, and milk analysis systems. However, combining all this information into one easy-to-use system remains a significant challenge. Problems, such as different formats, lack of standardization, and the absence of a national system to identify animals make it difficult for farmers to use their data efficiently. These challenges lead to missed opportunities for improving productivity, animal health, and farm operations. This paper brings together experts from research, farming, and industry to discuss solutions for integrating data on dairy farms. The group highlights the need for guidelines to ensure data can be shared and understood across systems, better tools to help farmers use their data, and stronger collaboration between industry and technology providers. Recommendations include educating farmers about data benefits, developing cost-sharing solutions, and using advanced technologies, like artificial intelligence, to improve decision-making. By addressing these issues, dairy farms can make better decisions, improve animal welfare, and achieve higher productivity and sustainability. These efforts will help create a future where farmers can fully harness the power of their data to meet growing food demands while protecting the environment.

## 1. Introduction

Data integration and analysis are pivotal in modern dairy farming, enabling farmers to optimize operations, enhance productivity, and achieve sustainability goals [1]. However, the dairy industry, like many other industries, faces substantial obstacles in merging diverse data sources and leveraging them for actionable insights [2]. The diversity of data types, formats, and sources, along with the rapid advancements in technology, presents unique challenges that require innovative solutions. This commentary aims to highlight these barriers, provide a comprehensive overview of discussions among a multidisciplinary group of experts, and propose pathways for advancing data integration. By bringing together insights from industry professionals, researchers, and farmers, we strive to foster dialogue and action toward building a more data-driven and sustainable dairy industry.

In dairy farming, data is generated from various sources, including herd management software, sensors, equipment, genetic evaluations, milk analysis and diagnostics laboratories [3], feeding systems, and environmental monitoring. Handwritten or unstructured records continue to be prevalent and problematic. Each of these data sources provides valuable insights, but integrating them into a cohesive system for comprehensive analysis and further insights remains a significant hurdle. Studies have shown that effective data integration can transform raw data into actionable insights, driving informed decision-making and operational efficiencies in agricultural systems, including dairy farming [1,4].

Data integration in the dairy industry faces challenges due to a lack of standardization, interoperability issues, and disparate data sources [5]. These barriers hinder seamless data sharing and effective utilization of information across systems.

For example, a dairy farm may use a body weight scale to measure cow weights, but the data are stored on a separate computer and are not accessible by the farm’s herd management software. The two systems may lack the capability to automatically share data because the body weight data file is in a format (e.g., .dif format with multi-line headers) that the farm management software cannot read. Integration would require a script to clean and prepare the data for import, as well as a shared network or drive to facilitate data transfer.

The absence of a single, uniform animal identification system in the US further complicates this issue. Unlike European countries and Canada, where such systems are widely implemented and facilitate seamless data integration across farms and systems, US producers struggle with fragmented animal IDs that limit data traceability and connectivity. Establishing a national animal identification system would provide a critical foundation for linking disparate data sources, enabling more effective data sharing and advanced analytics.

Without a real commitment from the farm’s leadership to allocate the necessary resources, this type of data integration often remains unaddressed. Standardized data exchange protocols and a unified identification system are essential to facilitate seamless data integration and enable the use of advanced analytical tools.

Furthermore, data ownership, privacy concerns, and the increasing monetization of data exchange add another layer of complexity. Farmers and companies often have reservations about sharing data due to intellectual property issues, competitive disadvantages, and the costs associated with data integration. The development of data exchange systems often incurs significant upfront costs, and ongoing data exchange fees between various parties can place additional financial burdens on farmers. Not only must farmers and companies pay for the initial development of an integration, but they are also frequently charged for continued data access and transfer. Developing clear guidelines and agreements on data ownership, usage rights, privacy, and cost structures is crucial to encourage data sharing while protecting stakeholders’ interests and ensuring fair monetization practices.

Infrastructure barriers, such as varying levels of IT infrastructure and connectivity, also impede data integration efforts. Without data integration, the role of advanced technologies, such as the Internet of Things (IoT), artificial intelligence (AI), and cloud computing may be limited. IoT devices can continuously collect real-time data from various sources, while AI and machine-learning algorithms can analyze this data to provide predictive and prescriptive insights. Cloud computing offers scalable storage and processing capabilities, enabling farms of all sizes to leverage advanced data analytics.

This commentary paper provides an overview of discussions from a multidisciplinary group of stakeholders, including industry experts, researchers, and practitioners. The group explored the importance of data integration, identified key barriers, and proposed strategies to overcome these barriers. Key areas of focus include technical challenges, data standardization, the role of advanced technologies, and the development of guidelines to facilitate actionable insights from diverse data sources.

By fostering a collaborative approach, involving various stakeholders and leveraging advanced technologies, the dairy industry can harness the power of integrated data to drive innovation and sustainability [6]. This paper aims to highlight the critical pathways forward for the dairy industry in overcoming data integration challenges and enhancing productivity and sustainability.

Our goal was to develop a commentary paper providing guidance and insights for the sector. The primary focus was to explore the challenges and opportunities in data integration and analysis. Objectives included identifying key challenges, proposing solutions, and developing a general framework for effective data management in the dairy industry. This collaborative effort underscores the need for a united approach to overcome technological, logistical, and economic barriers. To further refine our exploration, this commentary formulates a dual-objective approach: a scientific objective focused on advancing theoretical frameworks and methods for data integration and a practical objective aimed at developing actionable strategies to improve farm-level decision-making. These objectives help address research gaps in processing and leveraging large-scale data within dairy production systems on regional, national, and global scales.

## 2. Materials and Methods

This study was guided by two primary objectives: (1) a scientific objective to explore and develop theoretical frameworks for seamless data integration and standardized protocols across diverse dairy systems, and (2) a practical objective to design strategies and tools that enhance data-driven decision-making at the farm level. The overarching research problem addresses the fragmentation of data sources, lack of interoperability, and the absence of robust decision-support tools that limit the effective utilization of data in dairy farming.

The discussion meetings were both led and attended by the authors of this paper, who represent a multidisciplinary subgroup of the Dairy Brain Coordinated Innovation Network (CIN). The CIN is an initiative led by the University of Wisconsin–Madison to address data management challenges [4]. This subgroup, comprising researchers, academicians, industry professionals, extension specialists, and dairy farmers, brought diverse expertise and perspectives to the discussions. The participants’ dual role as facilitators and contributors ensured that discussions were deeply informed by their professional backgrounds and collective experience in addressing data integration challenges in the dairy industry.

Three structured meetings were held (followed by email interchanges), each focusing on specific aspects of data integration, including technical barriers, opportunities for standardization, and actionable pathways forward. A predefined agenda and guiding questions were used to steer the discussions. Examples of guiding questions included: “What are the primary barriers to data integration in dairy farming?” and “How can collaboration among stakeholders improve data sharing and usability?” The discussion segments were designed to cover various aspects of data integration, from technical challenges to strategic opportunities. 

In the final meeting, representatives from the International Committee for Animal Recording (ICAR; [7]) and AgGateway [8] were invited to provide insights on global standardization efforts. These representatives delivered short presentations outlining their work and its relevance to data integration in dairy farming, followed by a Q&A session and open discussion with the authors. Specifically, they presented their ongoing initiatives to create data exchange standards, discussed their approaches to addressing interoperability challenges, and clarified the applicability of their efforts within the dairy sector. Their contributions provided valuable context for the discussions, enhancing the group’s exploration of data integration solutions and highlighting potential pathways for collaboration.

The group used AI for notetaking, which allowed for accurate and efficient documentation of the meeting proceedings. The approach also emphasized the importance of confidentiality and adherence to a code of conduct, ensuring that all participants felt secure in sharing their insights and concerns.

## 3. Results

The discussions from this multidisciplinary group provided valuable insights into the current challenges and opportunities surrounding data integration and analysis in the dairy industry. By bringing together diverse perspectives from industry experts, researchers, and practitioners, the group identified critical issues hindering seamless data integration and proposed practical solutions to address them. The dialogue emphasized technical, infrastructural, and economic barriers, while also highlighting the role of advanced technologies and collaborative efforts in overcoming these challenges. The following key issues and proposed pathways forward summarize the outcomes of these discussions. The discussions were structured around these dual objectives, fostering a problem-solving approach that integrates both theoretical exploration and practical applications. By addressing the identified research problem, the group’s deliberations aimed to provide actionable insights while advancing knowledge on data integration in dairy systems.

### 3.1. Key Issues Discussed

#### 3.1.1. Importance of Data Integration for Actionable Insights

**Figure 1:**
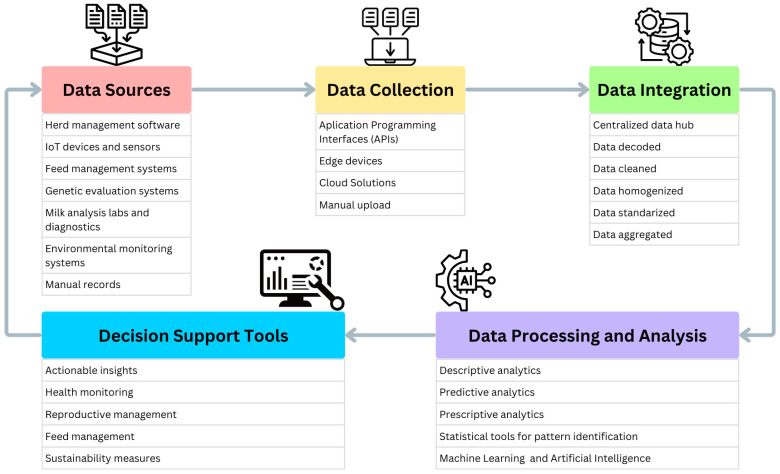
Framework for data integration in dairy farming: from collection to decision support.

The integration of diverse data sources is essential for deriving actionable insights and making informed decisions on dairy farms (Figure 1). Effective data integration combines data from herd management software, sensors, genetic evaluations, milk analysis and diagnostics laboratories, and feeding systems, enabling farmers to implement prescriptive tools that enhance productivity, health, animal welfare, profitability, and environmental sustainability. For example, farmers can use heat sensor data to identify which cows to breed or leverage somatic cell count (SCC) data from laboratories to detect mastitis and prioritize treatment. These practical applications demonstrate how integrated data supports daily herd management decision-making processes.

The data integration process involves several critical steps to ensure usability and consistency (Figure 1; [9]). Data decoding translates raw data into structured formats that are compatible with analytical tools. Data cleaning eliminates errors, duplicates, and inconsistencies, ensuring the integrity of datasets. Once cleaned, data homogenization standardizes units, formats, and terminologies across diverse sources, enabling seamless integration. This is followed by data standardization, which aligns metadata, coding systems, and frameworks to ensure compatibility across platforms. Finally, data aggregation consolidates multiple datasets, such as feed intake, milk production, and health records, into a unified repository, providing a comprehensive foundation for advanced analysis and decision-making. These steps collectively enhance the reliability, accessibility, and utility of data in dairy operations [9].

This holistic approach helps in identifying patterns and trends, leading to more efficient farm management practices. While challenges such as non-uniform data formats exist, systematic integration steps, like cleaning and standardization, can provide actionable insights, driving informed decision-making and operational efficiencies. Further complicating the issue, these data sources are often stored on different computers or systems and are not readily available online, making integration efforts even more difficult.

International cooperation is crucial in achieving seamless data integration. Global standardization efforts, exemplified by the International Committee for Animal Recording (ICAR; https://icar.org accessed on 15 December 2024) and AgGateway (https://aggateway.org accessed on 15 December 2024) provide foundational tools to enhance interoperability and improve data-sharing capabilities across systems [10]. These standards facilitate interoperability between different systems, allowing for the aggregation of data from various sources.

To deepen our understanding of global data standardization efforts and explore how these frameworks could enhance data integration in the dairy industry, we invited representatives from two leading organizations: ICAR and AgGateway. Both organizations presented their ongoing work in creating standards for data exchange, discussed their approaches to addressing interoperability challenges, and engaged in a Q&A session to clarify the applicability of their initiatives within the dairy sector. Their insights provided valuable context for our exploration of data integration solutions and highlighted potential pathways for collaboration.

The presentations highlighted the role of international organizations like ICAR and AgGateway in fostering standardized data exchange across agricultural sectors. ICAR’s work in developing independent guidelines and certification services, including animal identification and milk analysis, supports data standardization, which is essential for interoperability. AgGateway complements these efforts by facilitating data standards specifically for digital agriculture and providing resources like the Agricultural Data Application Programming Toolkit (ADAPT) for field operations and supply chain messaging. Together, these organizations help bridge gaps between proprietary systems, fostering collaboration and setting the foundation for effective data integration.

One major challenge is the differing business rules regarding the interpretation of data across regions, particularly between Europe and the US. These differences affect the applicability of data integration solutions and require careful consideration when developing global standards. For instance, European data handling practices often differ from those in the US, necessitating adaptations to ensure compatibility. For example, differences in date formats, such as mm/dd/yyyy used in the US versus dd/mm/yyyy commonly used in Europe, can lead to errors or misinterpretations when integrating data from these regions.

Another key aspect of data integration is its role in optimizing various operational aspects, such as feed management, milk production, and animal health monitoring. By integrating data from these diverse sources, farmers can gain comprehensive insights into their operations, enabling them to make more informed decisions. This integration supports the development of prescriptive tools that not only predict outcomes but also provide actionable recommendations, thereby improving overall farm efficiency. Furthermore, data integration can significantly enhance animal welfare, reproduction performance, and health. By combining health and production data, farmers can better monitor and manage the well-being of their herds. This integration allows for early detection of health issues, timely interventions, and continuous monitoring, ultimately leading to improved animal health and productivity.

The discussion also emphasized the importance of addressing technical challenges associated with data integration. These include ensuring data quality, managing large volumes of data, and maintaining data security and privacy. Internet connectivity was identified as a critical challenge, particularly in the US, where inconsistent or limited internet access can cause disruptions in data exchange. These breakdowns hinder real-time communication between systems, leading to inefficiencies and delays in managing day-to-day farm operations. The group highlighted the need for robust data management systems that can handle these challenges while providing reliable and accurate data for decision-making, even in low-connectivity environments.

Moreover, the potential for leveraging advanced technologies, such as artificial intelligence (AI) and machine learning (ML), through data integration was discussed. These technologies have the potential to enhance data analysis capabilities, providing deeper insights and predictive analytics that can further optimize farm operations. AI and ML can help in processing large datasets, identifying complex patterns, and generating actionable insights that are beyond the reach of traditional analytical methods.

**Figure 2:**
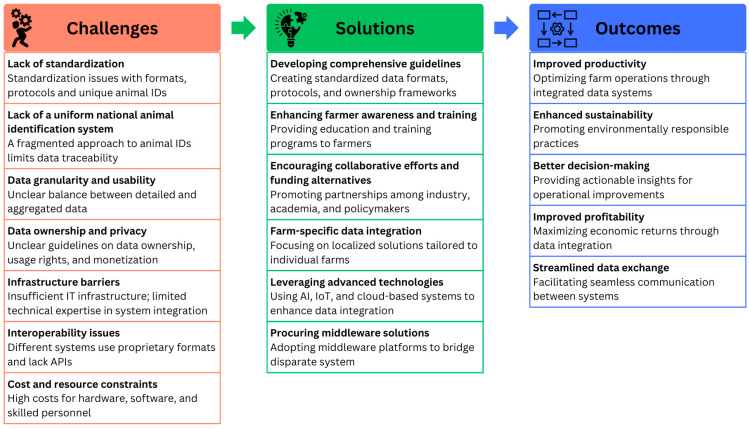
Challenges, proposed solutions, and expected outcomes.

In summary, the integration of diverse data sources is a critical factor in driving actionable insights and informed decision-making in dairy farming. While significant challenges exist, international cooperation, standardization efforts, and the adoption of advanced technologies can help overcome these barriers. By addressing these challenges, the dairy industry can harness the full potential of data integration to enhance productivity, profitability, animal welfare, and environmental sustainability. Figure 2 depicts the challenges, proposed solutions, and potential outcomes from the group discussions.

#### 3.1.2. Challenges in Data Integration

- Challenges in Data Exchange and Standardization

Discussions highlighted the difficulties in resolving technical and business-related issues in data exchange. The importance of standardization and data definition to improve data transfer was stressed. One of the primary challenges is the lack of standardized data formats and protocols across different systems. The group emphasized the need for global data exchange standards, referencing ongoing efforts by ICAR and AgGateway. Differences in data handling and interpretation across countries, particularly between Europe and the US, present unique challenges that must be addressed in data integration solutions.

This lack of standardization makes it difficult to integrate data from various sources, such as herd management software, milking parlor, sensors, and feeding systems. Different data systems often use proprietary formats, making interoperability a significant hurdle. The group emphasized the need for data exchange standards within the dairy industry, particularly noting the absence of a national animal identification system in the US. This lack of standardization makes it difficult to connect data across different systems.

In contrast, European countries and Canada benefit from having unique animal identifiers, which facilitate better data integration. These systems are mandatory, ensuring that animals are identified either at birth or prior to leaving the farm. Additionally, they include ID validation capabilities that help maintain data accuracy and traceability—features currently lacking in the US. Implementing similar systems in the US could significantly improve data connectivity, reduce errors, and streamline integration across platforms.

Additionally, there is an ongoing international effort through organizations like ICAR, involving manufacturers, maintenance, repair, and operations (MROs), and data record processing centers (DRPCs), to develop data exchange standards that enhance global interoperability. Differences in data handling and interpretation across countries present unique challenges. For example, variations in breeding practices and the (lack of) recording of dry dates between the US, Europe, and Canada highlight the need to incorporate these nuances in data integration solutions. Addressing these differences is crucial for developing robust and effective data integration frameworks that can be applied globally.

One major challenge discussed was the inconsistency in data standards and the lack of harmonization between regions [11]. For example, ICAR’s initiatives to standardize milk analysis and animal identification are crucial steps, but adoption varies by region. ICAR is widely recognized as a global leader in setting standards for data integration, particularly in the dairy industry (e.g., Animal Data Exchange Working Group; https://www.icar.org/index.php/technical-bodies/working-groups/animal-data-exchange-wg/ accessed on 15 December 2024), while AgGateway focuses more on data interoperability frameworks primarily in North America. However, there is currently no clear global authority or certification system that ensures compliance with these standards, adding to the complexity. Third-party verification mechanisms for data exchange could play a vital role in ensuring that companies follow established protocols and improve trust among stakeholders.

AgGateway’s data standards (AgGateway ADAPT Standard; https://adaptstandard.org/ accessed on 16 December 2024) also illustrate the difficulties in achieving industry-wide compliance, especially given that some standards apply primarily to North America. Ease of implementation remains a concern, as companies may face challenges in adopting these frameworks due to costs, technical expertise, or conflicting business priorities. This lack of harmonization across global dairy sectors limits data interoperability and complicates integration efforts. The inclusion of clear certification systems and improved resources to support adoption would help address these gaps, encouraging broader compliance.

- Data Granularity and Usability

The level of data granularity required varies depending on the application. For research purposes, highly granular data is critical as it allows for detailed analysis of specific variables and conditions, such as individual cow behaviors or minute-by-minute environmental changes. This granularity helps develop insights, refine models, and validate hypotheses with high accuracy. In contrast, farm management often benefits more from aggregated data that highlights actionable insights, such as average milk yield, overall herd health, or the identification of individuals or groups of cows in need of interventions, to make quick and effective decisions. Aggregated data is more manageable and directly applicable to optimizing daily operations.

Balancing the need for detailed data in research and simplified data in farm management is essential. Systems should be flexible enough to capture and store detailed data while also providing tools to aggregate and simplify them as needed. This ensures both researchers and farm managers can access data in the most useful form. Additionally, the cost and resource implications of data granularity must be considered, as collecting and managing highly granular data can be resource-intensive. Therefore, the benefits of detailed data must be weighed against the costs and feasibility of managing them.

- Data Ownership and Privacy

Concerns about data ownership, privacy, and monetization are critical challenges in facilitating data sharing. While companies may appear willing to share data, they often perceive limited value or face competitive disadvantages due to intellectual property concerns. This reluctance is especially evident with granular data, which include highly specific insights like individual cow performance metrics or sensor readings. Developing standardized contracts and agreements can address these issues by protecting stakeholders’ interests and clarifying data ownership, usage rights, and associated costs. Furthermore, ensuring that farmers retain control over their data and are adequately compensated for their use is essential to bridge the gap between the needs of academic research and commercial priorities.

- Infrastructure Barriers

Infrastructure barriers, including varying levels of IT infrastructure and connectivity, pose significant challenges. Computing devices, such as edge computing systems and central servers, are essential but require significant investment and expertise. In the end, each integration between two data sources inherently entails programming time which has a real cost at multiple levels. Also, whereas these devices generate valuable data, integrating these data into a cohesive system requires addressing compatibility and standardization issues. Network infrastructure, including reliable routers and switches, is crucial for seamless data transfer but can be difficult to establish and maintain, especially in rural areas. Additionally, firewalls are increasingly being implemented to secure systems, but they often create unintended barriers to data transfer. Larger dairy operations, in particular, may rely on local IT providers unfamiliar with the specific requirements of interconnected farm systems. This lack of expertise can result in breakdowns in data exchange, further complicating integration efforts. Backup power supply systems are also needed to prevent data loss and transfer during power outages. While cloud-based storage and computing offer benefits for data management and utilization, reliance on cloud solutions can introduce risks, such as data accessibility issues during internet outages, and cyber-attacks. Cybersecurity challenges further compound these infrastructure issues, as farm systems often lack robust defenses against cyberattacks, putting sensitive data and operational continuity at risk.

- Interoperability Issues

Different data systems often use proprietary formats, making interoperability a significant hurdle. The need for data exchange standards within the dairy industry was emphasized. This lack of standardization makes it difficult to connect data across different systems. Developing APIs (Application Programming Interfaces) and adopting common data exchange standards can mitigate this issue. Value propositions and incentives for data sharing throughout the value chain are required. The use of middleware solutions to bridge gaps between disparate systems was also suggested. Additionally, differences in how data are applied present another challenge. Business rules regarding data use can vary significantly between regions. For example, common practices in the US may not exist in Europe, where most major manufacturers are based. Interoperability issues arise when European manufacturers of milking robots, sensors, and monitoring systems, such as those for cow morphological features, body condition scoring, or rumination behavior, use proprietary data formats or communication protocols that are incompatible with US herd management software. These manufacturers often apply business rules tailored to European dairy management, causing compatibility issues in the US.

Furthermore, these challenges extend beyond the US and Europe, as data handling practices vary widely in regions such as Asia, Oceania, and Latin America. Differences in regulatory frameworks, infrastructure development, and the adoption of technological standards create additional barriers to achieving global data integration. Addressing these disparities will require international collaboration to ensure that standards and tools are flexible and adaptable to regional contexts.

One key issue identified by AgGateway is the difficulty of achieving consistency in data terminology and format, which often varies by region and even by manufacturer. For example, AgGateway pointed out that different software platforms may use unique terms or measurement units for the same data points, creating confusion and hindering effective data sharing. The organization’s efforts in developing a common data framework, such as through its ADAPT and AGIIS (ag Industry Identification System) tools, are steps towards resolving these issues by offering a standardized “language” for data that can be used across systems.

- Cost and Resource Allocation

Developing an in-house system to integrate data often requires substantial IT resources, which can be a significant barrier for many stakeholders, including researchers, smaller companies, and farms, particularly small to mid-sized operations. The high costs associated with hardware, software, and skilled personnel can be prohibitive, limiting the ability to leverage integrated data systems for improved decision-making and operational efficiency. While larger farms and companies may have the capacity to invest in these systems, smaller entities often lack the financial and technical resources to develop and maintain such solutions independently.

Even large companies may be reluctant to invest in development due to unclear or delayed return on investment. Despite the potential long-term benefits, the uncertainty surrounding immediate financial gains makes them hesitant to allocate resources to such development. This reluctance is often compounded by the need for ongoing maintenance and upgrades, further increasing the total cost of ownership. As a result, the adoption of integrated data systems remains challenging across the industry, necessitating alternative solutions such as cost-sharing models, government subsidies, or collaborative industry efforts to make these technologies more accessible.

### 3.2. Proposed Solutions and Pathways Forward

The proposed approach to data integration and analysis in dairy farming offers both direct and indirect benefits to farmers. Direct benefits include improved decision-making capabilities through the availability of prescriptive tools and actionable insights derived from integrated data systems. These tools can help optimize feed management, milk production, and animal health monitoring, leading to increased productivity, profitability, and sustainability on farms. For instance, farmers could use integrated systems to identify cows needing immediate health interventions or optimize feeding strategies to reduce costs and improve yields. Indirect benefits involve enhancing farmers’ knowledge and understanding of data-driven technologies and management practices. Through training programs, partnerships with universities, and awareness campaigns, farmers will gain the skills needed to leverage integrated data systems effectively, fostering long-term innovation and adaptation to emerging challenges in dairy production. By bridging the gap between technological solutions and practical farm management, this initiative aims to empower farmers to thrive in an increasingly data-driven agricultural landscape.

To achieve these benefits, a series of targeted solutions and pathways have been identified to address the key barriers to data integration and analysis. These solutions are designed to provide a structured and collaborative framework for overcoming the technical, logistical, and economic challenges faced by the dairy industry.

#### 3.2.1. Enhancing Collaboration and Developing Comprehensive Guidelines

The primary recommendation is to develop comprehensive guidelines for data integration and analysis. These guidelines should address:

- Standardized data formats and protocols to facilitate data exchange: ensuring consistency in file formats, APIs, and communication methods to enhance interoperability among systems;
- Collaborative frameworks and industry partnerships: encouraging partnerships not only among companies but also involving academic institutions, researchers, and farmers to foster innovation and inclusivity;
- Clear definitions of data ownership, usage rights, and data types: defining raw data (unprocessed, direct from sensors), processed data (cleaned or transformed data), and analyzed data (insights derived using analytical tools) to ensure common understanding;
- Novel frameworks for shared monetization: exploring equitable cost-sharing models to ensure stakeholders, including farmers, are not burdened with excessive costs for data exchange or integration;
- Robust data protection measures to ensure privacy and security: implementing safeguards to protect sensitive data and stakeholders’ interests.

Standardized protocols can reduce the need for customized interfaces and facilitate smoother data exchange across different systems. Implementing a national animal identification system can significantly enhance data integration. A unique identifier for each animal can enable seamless data tracking across various systems, improving data accuracy and connectivity. 

The discussion emphasized the importance of developing global compliance and standards for data integration and analysis in the dairy industry. The adoption of standardized data exchange frameworks, like those developed by ICAR and AgGateway, exemplifies how cross-industry collaboration can address key integration challenges. However, the existence of multiple standards can create confusion and hinder progress. The group noted the need for one clear governing body, such as ICAR, to lead these efforts, with organizations like AgGateway potentially aligning their frameworks with ICAR’s standards. Harmonizing data protection laws and regulations across regions can ensure consistent data management practices and facilitate international collaboration. Addressing legal and ethical considerations is critical for successful data integration, as these frameworks help safeguard stakeholder interests and foster trust among industry participants.

Effective data integration requires collaboration among various stakeholders. The discussion included several approaches to foster collaboration. Forming industry consortia and working groups can bring together stakeholders to develop and implement data integration standards. These groups can facilitate knowledge sharing, coordinate efforts, and advocate for common interests. Leveraging the expertise and resources of different stakeholders can accelerate progress. Public–private partnerships can provide funding, technical expertise, and policy support for data integration initiatives. Government agencies, industry associations, and academic institutions can collaborate on projects that benefit the entire dairy sector. Ensuring that these partnerships are transparent and inclusive is essential for their success.

There is a need for clear guidelines and agreements on data ownership, usage rights, data exchange costs, and privacy to facilitate data sharing. The discussion underscored the necessity of addressing data monetization and ensuring farmers are aware of the costs associated with data exchange. Developing standardized contracts and agreements that outline these aspects can facilitate data sharing while protecting stakeholders’ interests. The group proposed leveraging ICAR and AgGateway standards to enhance consistency and encourage broader adoption. Ensuring that farmers retain control over their data and are adequately compensated for their use is crucial. Additionally, the monetization of data exchanges needs to be addressed. While farmers should control who can access their data, they often have no control over the costs associated with data exchange, ultimately bearing these fees.

To address these challenges, both ICAR and AgGateway emphasized the need for collaboration across manufacturers, technology providers, and data platforms. ICAR is working on creating unique identifiers for devices, which will allow data from different manufacturers to be traced back to their source, helping researchers and farmers alike to standardize analyses. AgGateway also supports collaborative initiatives through projects like AGUS (AgGateway Unique Identifier System), a repository for unique identifiers, which promotes consistency across the supply chain. Such initiatives aim to make data integration more manageable and enable interoperability across systems.

Despite the competitive nature of the dairy industry, international cooperation with regard to data integration is both necessary and feasible. Organizations such as ICAR and AgGateway have already demonstrated success in creating standardized frameworks that enhance interoperability without compromising proprietary information. These standards allow companies and nations to innovate while ensuring a level playing field, particularly in areas of global concern such as sustainability, food safety, and climate change mitigation. Collaborative efforts on data integration can drive mutual benefits, including improved traceability, efficiency, and market access, while maintaining competitive boundaries.

#### 3.2.2. Enhancing Farmer Awareness and Training

Empowering farmers with knowledge about data integration and its benefits is crucial. Partnerships with universities were suggested to promote data analytics workshops for dairy farm owners and managers. Clear language should accompany equipment purchases, detailing data sharing policies and potential costs. Training programs and awareness campaigns can help farmers understand the value of integrated data systems and how to leverage them for improved decision-making. Partnerships with universities to promote data analytics workshops for dairy farm owners, managers, and consultants can further enhance their understanding and skills in this area. Additionally, dairy farmers need clear language accompanying the equipment they purchase, outlining how data can be shared, with whom, pass-through sharing of data, associated costs, and other relevant details. Many farmers are unaware of these aspects and often find out only after making their purchase.

Data security has emerged as a critical concern in the dairy industry, particularly as advanced technologies become more integrated into farm operations. Real-world incidents, such as the cyberattack of a milking robot in Switzerland, demonstrate the vulnerabilities faced by both large and small-scale operations. To address these risks, farmers must be equipped with practical cybersecurity knowledge and tools. Suggested strategies include adopting secure data transfer protocols, implementing firewalls, using reliable authentication methods, and ensuring regular software updates. Additionally, collaborative efforts between technology providers, industry associations, and academic institutions can help create affordable and accessible cybersecurity solutions tailored to the needs of dairy farmers.

The cooperation between farmers and dairy processors or cooperatives plays a pivotal role in effective data management. Farmers often have the greatest trust in their dairies, especially in cooperative structures where they are co-owners. Dairy processors or cooperatives can leverage this trust to offer targeted support in adopting and managing technical solutions. This includes providing advice on purchasing equipment and software, organizing training programs on their operation, and guaranteeing ongoing technical assistance.

#### 3.2.3. Encouraging Collaborative Efforts, Funding Alternatives, and User-Friendliness

Collaboration among stakeholders—farmers, industry players, academics, and policymakers—is vital. Creating platforms (and open access platforms) for ongoing dialogue and cooperation can help address emerging issues and refine data management practices.

Exploring cost-sharing models and funding opportunities to support data integration projects could alleviate this burden. Additionally, developing user-friendly tools that do not require extensive IT expertise can make data integration more accessible. The group emphasized the importance of collaborative efforts in addressing data integration challenges. International efforts, such as those by ICAR and AgGateway, were highlighted as models for establishing effective data exchange standards. However, the lack of a clear authority in this field remains a significant barrier, as it creates uncertainty and inefficiencies in standardization efforts. The group explored the idea of collaborating with ICAR to minimize duplication and maximize expertise. They discussed the distinction between technical and global issues addressed by ICAR and considered aligning efforts while maintaining valuable contributions.

#### 3.2.4. Localized Data Integration

Data integration could be separated into two levels: local integration for individual farms and global integration for researchers. The complexity of data integration in the dairy industry stems from several interconnected factors. Local integration, such as the Dairy Brain example, could focus on specific management strategies, whereas global integration could address broader research goals. Focusing on localized integration at the farm level can provide immediate benefits. By integrating data from various on-farm systems, farmers can gain valuable insights into operations, such as feed optimization, milk production, and animal health. This localized approach can also serve as a model for broader industry-wide integration efforts.

#### 3.2.5. Leveraging Advanced Technologies

Advanced technologies, such as the Internet of Things (IoT) devices, artificial intelligence (AI), and cloud computing, can facilitate data integration. However, it is crucial to balance the benefits of these technologies with considerations of cost, data accessibility, and security. Hybrid systems that combine local and cloud-based storage may offer a practical solution. IoT devices, such as sensors and smart tags, are increasingly used in dairy farming to monitor various parameters, including animal health, milk production, and environmental conditions. 

AI and machine learning (ML) algorithms can enhance data analysis, providing deeper insights and predictive capabilities. Developing AI/ML models tailored to dairy farming can optimize operations and improve decision-making. Ensuring these models are transparent and interpretable is crucial for gaining farmer trust and adoption. The rapid advancement of technology presents both opportunities and challenges for data integration in the dairy industry. 

The effectiveness of traditional statistical methods versus AI in data analysis was debated. The importance of understanding the problem before choosing a method was highlighted, along with the challenges of working with massive amounts of data and the potential need for machine learning.

#### 3.2.6. Role of Standardization Initiatives

Standardization initiatives, such as those by ICAR and AgGateway, play a pivotal role in addressing data integration challenges in the dairy industry. ICAR has developed independent guidelines and certification services, including animal identification and milk analysis, to foster interoperability and consistency across platforms. Similarly, AgGateway promotes data standards tailored to digital agriculture and provides tools like the ADAPT toolkit for streamlining field operations and supply chain messaging. These initiatives serve as critical examples of how collaborative, cross-industry efforts can enable seamless data exchange while respecting proprietary concerns. Emphasizing their approach highlights the importance of fostering global cooperation and adopting scalable frameworks to address the growing complexities of data integration.

### 3.3. Future Directions and Research Opportunities

Several future directions and research opportunities were identified. The potential of AI and machine learning in enhancing data integration and analysis was discussed, with suggestions to explore the use of large language models (LLMs) for automating data queries and integration tasks. Research and development efforts should focus on creating scalable data integration solutions that can be adapted to farms of different sizes and complexities. Ensuring that these solutions are cost-effective and user-friendly is critical for widespread adoption. Advancing the use of AI and ML in dairy farming presents significant opportunities. Research should explore the development of models that can predict and prescribe actions based on integrated data. 

Future efforts should focus on practical applications and farmer engagement to advance data integration in dairy farming. Pilot studies on farms can test the feasibility of proposed solutions, while surveys and participatory workshops with farmers and stakeholders can identify specific challenges, expectations, and opportunities for improvement. These initiatives, combined with tailored training programs, will bridge the gap between theoretical discussions and real-world implementation, driving the adoption of data systems that enhance productivity, profitability, and sustainability.

Collaborating with AI experts and data scientists can enhance these efforts. Improving traceability and consumer benefits, and sustainability through data integration can benefit the entire dairy supply chain. Research should investigate how integrated data can support sustainable practices, enhance food safety, and meet consumer demands for transparency. The need for standardized ways of communicating data between systems was highlighted, which could be beneficial for both local and global integration efforts. While many companies are moving towards cloud-based storage for easier data analytics, local data storage remains critical for uninterrupted farm operations. Implementing a national animal ID system, farm scale, and specific data exchange needs should be further explored in future research.

Looking ahead, the integration of AgGateway’s ADAPT tool with ICAR’s guidelines offers promising opportunities to harmonize data sources across livestock and crop production. Future research should focus on how such standards can be applied to emerging technologies, such as AI-driven sensors and automated feeding systems, to support real-time decision-making. Engaging dairy producers and stakeholders in these conversations will be key to refining and implementing these standards on a global scale.

### 3.4. Limitations

While the proposed approach to data integration and analysis in the dairy industry offers promising pathways forward, several limitations must be acknowledged. Infrastructure barriers, such as inconsistent internet connectivity and insufficient IT resources, particularly in rural areas, remain significant obstacles to seamless data integration. Additionally, the lack of global standardization in data formats, protocols, and animal identification systems continues to challenge interoperability across platforms and regions. The high costs associated with implementing data integration systems, including hardware, software, and skilled personnel, can deter smaller farms and stakeholders from adopting these solutions. Furthermore, concerns about data ownership and privacy, as well as the monetization of data exchanges, add another layer of complexity. Achieving broad adoption will require technological innovation, sustained collaboration among stakeholders, significant policy support, and ongoing research to address these multifaceted barriers. These limitations highlight the need for a phased and flexible approach that can adapt to varying levels of infrastructure, technical capacity, and stakeholder readiness across the industry.

## 4. Conclusions

Data integration and analysis are critical for the advancement of the dairy industry. Addressing the challenges in data integration requires a collaborative approach, involving the development of comprehensive guidelines, technological advancements, and stakeholder engagement. By fostering an environment of transparency and cooperation, the dairy industry can harness the power of integrated data to drive innovation and sustainability.

Data integration and analysis are critical for the advancement of the dairy industry. These processes enable dairy farmers to make informed decisions, optimize operations, and enhance productivity, ultimately contributing to the overall sustainability of the industry. Addressing the challenges in data integration requires a collaborative approach, involving the development of comprehensive guidelines, technological advancements, and stakeholder engagement.

A collaborative approach is essential in overcoming the multifaceted challenges associated with data integration. Stakeholders, including farmers, industry experts, technology providers, and researchers, must work together to establish common goals and share best practices. By fostering an environment of transparency and cooperation, the dairy industry can harness the power of integrated data to drive innovation and sustainability. This collective effort will help in identifying and addressing the technical, logistical, and financial barriers that impede effective data integration.

The development of comprehensive guidelines is a cornerstone in facilitating data integration. These guidelines should encompass standardized data formats and protocols, data ownership and privacy policies, and clear procedures for data sharing. Standardization is particularly important as it ensures interoperability between different systems and platforms, allowing for seamless data exchange. International efforts, such as those by ICAR and AgGateway, play a crucial role in establishing these standards and promoting global compliance.

Technological advancements are pivotal in addressing the complexities of data integration. The adoption of advanced technologies, such as the Internet of Things (IoT), artificial intelligence (AI), and cloud computing, can significantly enhance data collection, processing, and analysis capabilities. IoT devices can provide real-time data on various aspects of dairy farming, while AI and machine-learning algorithms can analyze this data to generate actionable insights. Cloud computing offers scalable storage solutions and facilitates remote data access, making it easier for farmers to leverage integrated data systems.

Stakeholder engagement is vital in ensuring the successful implementation of data integration initiatives. Farmers, in particular, need to be empowered with the knowledge and tools required to utilize integrated data systems effectively. Training programs and awareness campaigns can help farmers understand the benefits of data integration and how to leverage it for improved decision-making. Additionally, collaborative efforts between universities and industry can promote the development and dissemination of data integration technologies and practices.

Addressing legal and ethical considerations is also critical for successful data integration. Clear guidelines on data ownership, usage rights, and privacy must be established to protect stakeholders’ interests. Developing standardized contracts and agreements can facilitate data sharing while ensuring that farmers retain control over their data and are adequately compensated for their use. This approach will help build trust among stakeholders and encourage wider participation in data-integration initiatives.

Furthermore, there is a need to address the infrastructural barriers that hinder data integration, particularly for smaller farms. Investments in IT infrastructure, including reliable network connectivity and robust computing systems, are essential to support data-integration efforts. Exploring alternative funding models, such as cost-sharing arrangements and government subsidies, can help make these investments more accessible to small and medium-sized farms.

Data integration and analysis hold immense potential for advancing the dairy industry. By adopting a collaborative approach that involves the development of comprehensive guidelines, leveraging technological advancements, and ensuring stakeholder engagement, the industry can overcome the challenges associated with data integration. This will pave the way for enhanced productivity, profitability, and sustainability in dairy farming. The future of the dairy industry lies in its ability to harness the power of integrated data, and collective efforts in this direction will yield significant benefits for all stakeholders involved.

The insights from ICAR and AgGateway reinforced the need for a unified approach to data integration, where internationally recognized standards are adopted across the dairy industry. As data continue to grow in complexity and volume, aligning with these standards will be essential for achieving interoperability and maximizing the value of data analytics in dairy production. By following these guidelines, the dairy industry can build a resilient, data-driven foundation that supports productivity, animal welfare, and environmental goals.

This commentary identifies critical barriers to data integration in dairy farming, including the lack of standardization, infrastructure limitations, and concerns over data ownership and privacy. To address these challenges, the paper highlights the importance of establishing comprehensive data integration frameworks that enhance interoperability and provide actionable insights for decision-making. The scientific objective is to advance knowledge on scalable and inclusive data integration solutions, while the practical objective focuses on improving farm-level operations, sustainability, and profitability. Addressing these issues offers opportunities for future research and innovation to build a resilient, data-driven dairy industry.

## Data Availability

Not applicable. This commentary paper does not involve the generation or use of any datasets. It is based solely on the opinions, expertise, and discussions of the authors.
